# Potential Therapeutic Targets and Promising Agents for Combating NAFLD

**DOI:** 10.3390/biomedicines10040901

**Published:** 2022-04-14

**Authors:** Atsushi Umemura, Seita Kataoka, Keiichiro Okuda, Yuya Seko, Kanji Yamaguchi, Michihisa Moriguchi, Takeshi Okanoue, Yoshito Itoh

**Affiliations:** 1 Department of Pharmacology, Kyoto Prefectural University of Medicine, 465 Kajii-cho, Kamigyo-ku, Kyoto 602-8566, Japan; 2 Molecular Gastroenterology and Hepatology, Graduate School of Medical Science, Kyoto Prefectural University of Medicine, 465 Kajii-cho, Kamigyo-ku, Kyoto 602-8566, Japan; s1120@koto.kpu-m.ac.jp (S.K.); k-okuda@koto.kpu-m.ac.jp (K.O.); yuyaseko@koto.kpu-m.ac.jp (Y.S.); ykanji@koto.kpu-m.ac.jp (K.Y.); mmori@koto.kpu-m.ac.jp (M.M.); yitoh@koto.kpu-m.ac.jp (Y.I.); 3 Department of Gastroenterology and Hepatology, Saiseikai Suita Hospital, Suita 564-0013, Japan; okanoue@suita.saiseikai.or.jp

**Keywords:** NAFLD, NASH, HCC, honokiol, HSC

## Abstract

Nonalcoholic fatty liver disease (NAFLD), including nonalcoholic steatohepatitis (NASH), is a growing cause of liver cirrhosis and liver cancer worldwide because of the global increases in obesity, dyslipidemia, hypertension, and type 2 diabetes mellitus. Contrary to the advancements in therapies for viral hepatitis, effective treatments remain unestablished for patients with NAFLD. NAFLD, including NASH, is characterized by steatosis, inflammation, hepatic necrosis, and fibrosis. Despite our understanding of its pathophysiology, there are currently no effective treatments for NAFLD. In this review, we provide an update on the known pathophysiological mechanisms involved in the development of NAFLD and the role of hepatic stellate cells, and summarize the potential therapeutic agents, including natural products, for NAFLD.

## 1. Introduction

Nonalcoholic fatty liver disease (NAFLD), including nonalcoholic steatohepatitis (NASH), reportedly affects the health status of approximately one in four people worldwide, according to a recent meta-analysis [1]. The prevalence of this disease is projected to increase in the future due to the growing numbers of people with obesity, dyslipidemia, hypertension, and type 2 diabetes (T2DM). NAFLD is one of the most common causes of liver failure necessitating transplantation in the U.S. [2]. Due to the global increase in metabolic syndrome and the advancements in therapies for viral hepatitis, NAFLD is expected to become the most common cause of liver cirrhosis and liver cancer in the near future, surpassing viral etiologies [3].

NAFLD can be categorized histologically into nonalcoholic fatty liver (NAFL) and NASH. While NAFL is defined as the presence of hepatic steatosis without necroinflammation, the key pathological features for the diagnosis of NASH are inflammation, necrosis, fibrosis in the liver, the ballooning degeneration of hepatocytes, and hepatic steatosis [4]. Without therapeutic interventions, 7–30% of NAFL cases may progress to the necroinflammatory form, NASH [1,3]. In a recent meta-analysis of more than 4000 patients with NAFLD, liver fibrosis was tightly associated with all-cause mortality as well as liver-related mortality [1,5]. Therefore, liver fibrosis is the most important factor contributing to the prognosis of patients with NAFLD and has been a major focus of the emergence of targeted therapies for this disease [6]. A prospective study yielded the same results and concluded that fibrosis stage is positively associated with risks of liver-related complications and death [7]. NASH and advanced fibrosis need to be identified in order to prevent the related complications.

Nevertheless, there are increased risks of all-cause mortality, liver-related mortality, and cardiovascular events in patients with NAFLD compared with the general population [1,5,8]. Apart from diet and weight loss, there is no established treatment for NAFLD, despite the various clinical trials that have been conducted. Of note, liver fibrosis can serve as a surrogate endpoint for the outcomes in clinical trials. Although potential pharmacotherapeutic agents have been tested in clinical trials, most of them have failed to achieve the expected outcomes of steatohepatitis resolution or reversal of fibrosis. Now there is a large unmet need to gain a deeper understanding of the underlying mechanisms in NAFLD and to identify and develop effective treatment options for the benefit of patients. Therefore, it is important to establish therapeutic agents and preventive interventions with the potential to reduce the risk of the progression of NAFLD, particularly approaches that combat liver cirrhosis and hepatocarcinogenesis.

In this review, we discuss NAFLD pathophysiology and the factors related to the progression from NAFL, which is a simple steatotic stage, to NASH, which is characterized by necroinflammation, fibrosis, and ballooning degeneration. We also discuss the potential for treatment and prevention.

## 2. Factors Associated with NAFLD Pathophysiology

NAFLD is associated with obesity, dyslipidemia, hypertension and T2DM, which are clinical features of metabolic syndrome and are associated with insulin resistance and adipose tissue dysfunction [1,9]. Obesity is considered the most important factor influencing the development of fatty liver [10]. The severity of hepatic steatosis is significantly correlated with body mass index, visceral fat thickness and insulin resistance. The incidence of NAFLD is significantly higher among patients with T2DM compared with the general population [11]. In addition, T2DM increases the risk of cirrhosis and HCC among patients with NAFLD [12].

The gut microbiota is associated with advanced fibrosis in NAFLD patients. During the progression of NAFLD to advanced fibrosis, the abundance of the Proteobacteria phylum significantly increases, while that of the Firmicutes phylum decreases [13]. In patients with NAFLD-related cirrhosis, the gut microbiota profile and systemic inflammation are significantly correlated and are involved in hepatocarcinogenesis [14].

Genetic predisposition also has an important impact on the development of NAFLD. In 2008, for the first time, genome-wide association studies reported the I148M single nucleotide polymorphism (SNP; rs738409, C > G) in the patatin-like phospholipase domain containing protein 3 (*PNPLA3*), which is a susceptibility gene involved in the development of NAFLD [15]. Adiponutrin encoded by *PNPLA3* is expressed on adipose cell membranes and promotes lipase activity, predominantly during lipid metabolism [16]. PNPLA3 I148M increases the activity of lysophosphatidic acid acyltransferase, which regulates hepatic liposynthesis, a major determinant of lipogenesis in the liver. PNPLA3 I148M is widely involved in the pathogenesis of NAFLD and contributes to fibrosis progression and hepatocarcinogenesis [17,18,19]. Transmembrane 6 superfamily member 2 (TM6SF2) activity is required for normal VLDL secretion, and impaired TM6SF2 activity causally contributes to NAFLD and is associated with steatosis, inflammation and fibrosis [20,21]. A splice variant (rs72613567) of the 17-β-hydroxysteroid dehydrogenase 13 gene (*HSD17B13*) has been associated with increased steatosis, although it decreases inflammation and ballooning degeneration via its inherent hepatic retinol dehydrogenase activity. This rs72613567 variant attenuates the progression of fibrosis and may reduce the risk of NAFLD-related cirrhosis [22]. Other SNP variants, such as glucokinase gene regulator (*GCKR*)*,* GATA zinc finger domain containing 2A (*GATAD2A*), membrane bound O-acyltransferase domain-containing *7* (*MBOAT7*)*,* and phosphatidylethanolamine N-methyltransferase (*PEMT*)*,* may also be implicated in the pathophysiology of NAFLD [23,24,25,26]. Notably, the genetic alterations and their consequences can be potential targets for the treatment of NAFLD. Currently, a clinical study of an antisense medicine AZD2693, which was designed to inhibit the production of PNPLA3 protein, is ongoing to investigate its effects on patients with NASH, fibrosis stage 0 to 3, who are homozygous for the PNPLA3 148M risk allele (ClinicalTrials.gov identifier: NCT04483947).

## 3. Pathophysiology of NAFLD

Notably, the most important factor for the disease progression of NAFLD and for the development of its treatment is liver fibrosis. Various pathways have been implicated in fibrosis progression in patients with NAFLD. Clinical studies have shown that factors such as older age, insulin resistance, diabetes mellitus, circulating adipokines, and serum ferritin levels are predictive of fibrosis progression [27]. Furthermore, multiple factors are proposed to interact with each other to increase oxidative stress, induce inflammation, and contribute to fibrosis progression.

A mechanism of hepatic fibrosis is excessive production of collagen via the activation of hepatic stellate cells (HSCs). Normally quiescent HSCs, which are essentially vitamin-A-storing cells, transdifferentiate into activated HSCs in injured liver tissues and differentiate into proliferative fibrogenic myofibroblast-like cells that, via constitutive and induced expression, generate cytokines and growth factors such as PDGF and TGF-β [27,28,29,30]. Activation of HSCs via extracellular signals from resident and inflammatory cells, including macrophages, hepatocytes, endothelial cells, T cells, B cells, and other immune cells, is also an important factor [27].

Autophagy is a metabolic process that degrades and recycles intracellular organelles and their constituents, and it has many roles in human pathological conditions, including liver diseases. Recent studies have suggested a role of autophagy in the development of NAFLD [31] and have shown that autophagy can induce lipid droplet degradation in hepatocytes, a process called lipophagy [32]. Increased autophagic flux was observed in activated HSCs, and accordingly, treatment with bafilomycin A1—an autophagy inhibitor—was shown to suppress the expression of profibrotic markers and inhibit cellular proliferation in HSCs [33]. Understanding the mechanism of autophagy in the activation process of HSCs may lead to new therapeutic strategies for liver fibrosis.

As activated HSCs play a pivotal role in progressive hepatic fibrogenesis [34], they represent a potential target for antifibrotic therapeutics. Despite the numerous advances made towards understanding the pathogenesis of liver fibrosis, to date there are no specific antifibrotic drugs that have received U.S. Food and Drug Administration (FDA) approval, and effective treatments are lacking. The major challenge to overcome in the treatment of liver fibrosis pertains to its slow rate of progression to cirrhosis. Chronic mechanisms give rise to modifications of liver vascularization, the composition of the extracellular matrix, and drug metabolism. The currently available therapies to treat these conditions remain inadequate [27], and there is an urgent unmet need for new clinically effective and safe therapies.

## 4. Drugs

There is an increasing demand for new clinically effective and safe therapies for the treatment of NAFLD. For the improvement of NAFLD, the treatments of concomitant lifestyle-related diseases, especially T2DM, dyslipidemia, and hypertension, are the most important, and anti-fibrotic agents are needed. Thiazoline derivatives are thought to improve insulin resistance and lipid metabolism by acting as agonists of peroxisome proliferator-activated receptor (PPAR)-γ, regulating fatty acids and inflammatory adipokines secreted by adipocytes, and promoting adiponectin secretion. According to a meta-analysis, treatment with thiazoline derivatives improves the histological findings of NAFLD, including fibrosis, by reducing lipogenesis, inflammation, and ballooning degeneration. However, the effects of these changes on preventing progression to cirrhosis and improving prognosis remain to be verified [35].

Glucagon-like peptide-1 (GLP-1) receptor agonists and dipeptidyl peptidase-4 inhibitors, which inhibit an enzyme that degrades GLP-1, are drugs related to incretins that promote insulin secretion from pancreatic beta cells. GLP-1 suppresses motility in the upper gastrointestinal tract (e.g., the stomach) and has been shown to reduce weight gain by inducing a feeling of satiety in patients with T2DM [36]. These same effects have been investigated for therapeutic use in patients with NAFLD. Two randomized phase II trials of GLP-1 analogs for treatment of NAFLD showed histological resolution, although the effects on fibrosis stage remain to be investigated [37,38]. Conversely, the results of a meta-analysis of dipeptidyl peptidase-4 inhibitors were mixed, and no consensus was reached [39]. The results of clinical trials evaluating GLP-1 analogs for the treatment of NAFLD are expected in the near future, and the feasibility of using oral drugs, in addition to injectable drugs, is also being evaluated.

Vitamin E is a free radical scavenger that captures reactive oxygen species and inhibits lipid and protein oxidation; its efficacy in NAFLD was confirmed in a 2015 meta-analysis that examined serum liver enzymes and liver histology [40]. However, the clinical utility and safety of long-term vitamin E administration await future verification.

Sodium glucose co-transporter 2 (SGLT2) inhibitors block the reabsorption of glucose filtered by glomeruli in the proximal renal tubules and lower blood glucose in an insulin-independent manner. In terms of treatments for NAFLD, many reports have indicated that SGLT2 inhibitors improve liver function and decrease liver steatosis, rendering this class of drugs promising [41]. Improvements in inflammation, ballooning degeneration, and fibrosis have been reported only in a small number of patients and over a relatively short period of time. Therefore, studies elaborating on the usefulness of long-term administration of SGLT2 inhibitors in terms of improving liver histology are needed.

Biguanides, particularly metformin, are first-line antihyperglycemic drugs for the treatment of T2DM [42]. Pharmacological metformin doses improve mitochondrial respiration by increasing mitochondrial fission via AMPK activation, leading to suppression of glucose production in the liver [43]. Although numerous studies have been conducted, the effects of metformin on liver histology and serum liver enzymes have not been convincing [44,45]. However, since both clinical and basic studies have reported that carcinogenesis can be inhibited by metformin, large-scale long-term randomized trials of metformin, with and without concomitant drugs, are desirable [46].

Hydroxymethylglutaryl-CoA reductase inhibitors have been widely reported to improve serum lipid and liver enzyme concentrations, although no accurate histological evaluations have been conducted [47]. There have been several reports investigating the efficacy of ezetimibe in NAFLD, although no definitive conclusions have been reached [48]. Regarding ursodeoxycholic acid, no effect of regular doses on NAFLD has been shown [49], although it may be useful at high doses [50].

The involvement of HSCs in the development of NAFLD and fibrosis has been shown in many basic and clinical studies, and therapeutic interventions targeting angiotensin II receptors expressed on HSCs have also been investigated. Angiotensin II receptor blockers and angiotensin-converting enzyme inhibitors have been shown to reduce liver fibrosis and inflammation in patients with NASH, thereby improving liver histology and liver function [51,52,53]. Many patients with NASH have concomitant hypertension, and angiotensin II receptor blockers and angiotensin-converting enzyme inhibitors are widely used as antihypertensive drugs.

## 5. Therapeutic Potential of Honokiol in NAFLD

### 5.1. Honokiol, an Anti-Fibrotic Agent That Modulates Autophagy

Natural compounds and their derivatives also serve as potential agents for developing new drugs for NAFLD. Honokiol (HNK) is a natural, bioactive compound extracted from the species *Magnolia grandiflora* that has been used extensively as a component of herbal medicines [54,55]. HNK was shown to exert suppressive effects in a rat model of liver fibrosis [56,57], although the mechanism of action was not elucidated or explored in mouse livers. In a renal fibrosis study, HNK suppressed progressive fibrosis via inhibition of the TGF-β1/SMAD signaling pathway in HSCs [58]; this may indicate a mechanism central to the antifibrotic effects of HNK.

Although the definitive mechanisms by which HSC activation is involved in the progression of chronic liver injury to cirrhosis remain elusive, it is conceivable that increased autophagic flux plays a central role [59,60,61]. This notion raises the possibility that partial inhibition of HSC activation following treatment with autophagy inhibitors provides a novel therapeutic approach to combat liver fibrosis. Indeed, we found that HNK suppressed autophagy in HSCs [62]. The selective decrease in autophagic activity in HSCs has demonstrated potential as a therapeutic approach for patients with fibrotic diseases, including liver fibrosis [59,60,61]. The idea that HNK administration inhibits autophagy in HSCs aligns with the aforementioned proposed mechanism underlying the inhibitory effects of HNK on fibrosis progression.

**Figure 1:**
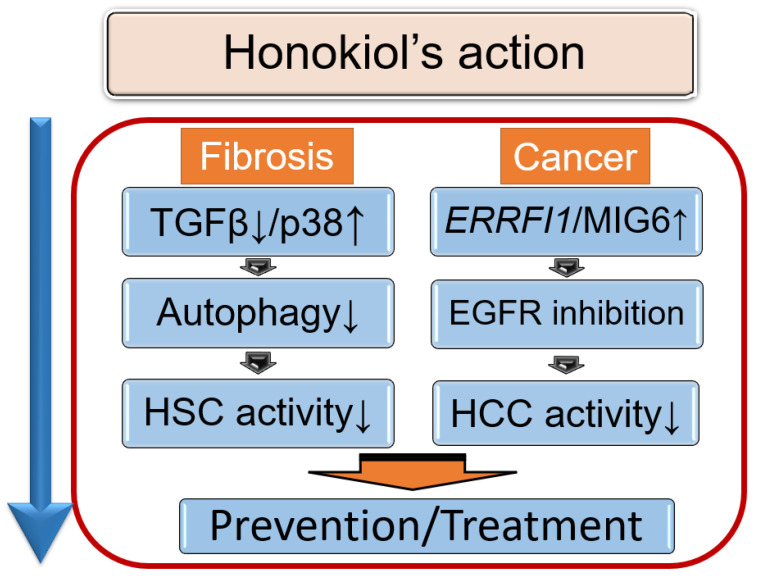
The actions of honokiol.

Overall, HNK has several intriguing mechanisms of action [62]. It has been associated with suppression of the TGF-β1/SMAD signaling pathway and autophagy. Studies have confirmed at least partial attenuation of HSC activation via autophagy inhibition (Figure 1). Other studies focused on the ability of HNK to downregulate autophagy and attenuate HSC activation via induction of p38 activation, a pathway independent of TGF-β1/SMAD signaling [59,60,61]. In addition to its antifibrotic effects in the liver [62], HNK also exhibits antitumor properties [63]. Its frequent use as a component of herbal medicines, together with its therapeutic potential as a treatment for liver fibrosis and its sequelae, liver cirrhosis, and liver cancer, warrants further investigation of the potential clinical applications of HNK.

A schematic model of the actions of honokiol (HNK). HNK suppresses TGFβ, leading to a reduction in hepatic stellate cell (HSC) activity. In addition, HNK activates p38 and then inhibits autophagy, which also suppresses HSCs. HNK activates ERBB receptor feedback inhibitor 1 gene (*ERRFI1*) expression, encoded by *ERRFI1*, leading to EGFR inhibition in hepatocellular carcinoma (HCC). In NAFLD-afflicted livers, EGFR signaling is upregulated, and exposure to HNK prevents HCC induction via EGFR downregulation. As a result, HNK exerts anti-fibrotic and anti-tumor effects.

### 5.2. Honokiol and Its Anti-Tumor Effects

NAFLD, specifically NASH, is associated with an increased risk of hepatocellular carcinoma (HCC). It is also known that NAFLD-associated HCC can develop in patients with or without cirrhosis [64]. The rate of carcinogenesis in patients with NAFLD depends on the characteristics of the population investigated and the research methods employed. With that caveat in mind, approximately 0.04% of patients per year with NAFLD and approximately 2–3% of patients per year with NASH cirrhosis develop HCC [1,65,66]. Although these rates are expected to be lower than the rate of HCC development from viral hepatitis, patients who are male, have cirrhosis, or are elderly have a higher HCC risk [67]. In addition, with regard to overall cases of HCC in the context of NAFLD, the carcinogenesis rate is not high, but due to the large population, cancer development in non-cirrhotic livers is not uncommon [67]. NAFLD has complex and heterogenous root causes, rendering it challenging to develop broadly appropriate NAFLD-HCC drugs, particularly prophylactic pharmaceuticals.

The EGFR signaling pathway plays a key role in liver regeneration following acute and chronic liver damage, and in cirrhosis and HCC [68]. EGFR is a critical regulator of the regenerative process in the liver and of hepatocyte proliferation [69,70]. EGFR inhibition was recently demonstrated to halt the development of liver steatosis and liver injury in a murine model of NAFLD [71,72]. In NAFLD-afflicted livers, EGFR signaling is upregulated, and exposure to HNK prevented HCC induction via EGFR downregulation [73].

The antitumorigenic activity of HNK is thought to be exerted via activation of GR, which accumulates in the nuclei of HNK-treated HCC cells [73]. GR activation stimulates mitogen-inducible gene-6 (MIG-6) expression. MIG-6, which is encoded by the ERBB receptor feedback inhibitor 1 gene (*ERRFI1*), is a cytoplasmic protein that functions as a tumor suppressor in lung, skin, breast, pancreatic, and ovarian malignancies [74,75,76]. Moreover, MIG-6 is reportedly downregulated in human HCC, and this is correlated with increased expression of EGFR [77]. Given that HNK induces GR translocation to the nucleus, it may be conceivable that MIG-6 expression and subsequent EGFR degradation mediate the ability of HNK to interfere with the progression from NASH to HCC [73]. In addition, *ERRFI1* expression in patients with NAFLD decreased in parallel with disease progression, characterized by severe inflammation, strong ballooning degeneration, and advanced fibrosis. Advanced NASH exhibited decreased *ERRFI1* and increased *EGFR* expression, which supports the clinical importance of the MIG-6/EGFR axis in NASH-induced HCC development. Therefore, induction of *ERRFI1* expression, in response to GR activation by HNK, could be a strategy for HCC prevention in patients with chronic liver diseases, particularly those with NAFLD [73].

Taken together, the evidence thus far indicates that the GR/MIG-6 axis is implicated in the degradation of EGFR to retard the progression of NASH to HCC. HNK activates MIG6 expression, leading to EGFR inhibition, and exerts anti-tumor effects and anti-fibrotic ones.

## 6. Future Therapeutic Drugs in Development

### 6.1. Pharmacological Agents in Clinical Trials

Current clinical trials investigating the use of pharmacological agents for the treatment of NAFLD (Table 1) tend to focus on GLP-1 receptor agonists, SGLT2 inhibitors, or PPAR agonists. For example, the efficacy of the GLP-1 receptor agonist semaglutide in the treatment of NAFLD has been investigated intensively [38]. Furthermore, molecules with dual activity against glucose-dependent insulinotropic polypeptide (GIP) receptor and GLP-1 receptors, such as tirzepatide [78] and BI456906 (ClinicalTrials.gov identifier: NCT04771273), may offer better blood glucose and weight control compared with currently available GLP-1 receptor agonists.

In addition, the PPARα/δ/γ agonist lanifbranor [79] is a representative pan-PPAR agonist in clinical development for the treatment of NAFLD. Other applicable PPAR agonists target only one or two PPAR isoforms for activation. For example, pemafibrate [80] is a PPARα-specific agonist, and saroglitazar [81] is a dual PPARα/γ agonist.

While the focus has been on these more typical targets for the treatment of NAFLD, this section introduces some other candidate drugs under investigation, along with their proposed targets and mechanisms. One such drug is obeticholic acid [82], which is an agonist of the farnesoid X receptor (FXR). The activation of FXR by obeticholic acid is involved in the modulation of not only bile acid metabolism but also lipid and carbohydrate metabolism, and this activation also influences insulin sensitivity. The improvements in NASH and hepatic fibrosis by obeticholic acid were reported in the phase 2 study FLINT [83] and are being investigated in the ongoing phase 3 study REGENERATE to assess clinical outcomes [82].

Thyroid hormone receptor β (THR-β) is highly expressed in hepatocytes and is responsible for regulating the metabolic pathways in the liver that are frequently impaired in NAFLD. Animal studies have shown that THR-β has important roles in reducing triglyceride and cholesterol levels, improving insulin sensitivity, promoting liver regeneration, and reducing apoptosis. A THR-β agonist, resmetirom [84], was shown to reduce elevated liver enzymes in NASH patients in a phase 2 study. The THR-β selectivity of resmetirom is correlated with an enhanced safety profile compared with those of non-selective agents. Currently, two phase 3 studies of resmetirom in patients with NASH and liver fibrosis, MAESTRO-NASH and MAESTRO-NAFLD-1, are ongoing.

Stearoyl-CoA desaturase (SCD) 1, which catalyzes the rate-limiting step in the biosynthesis of monounsaturated fatty acids, is considered a promising target for the treatment of NAFLD. Aramchol [85], a partial inhibitor of hepatic SCD1, was found to improve liver histology and enzymes in the phase 2 trial ARREST, although the primary end points did not reach the prespecified levels of significance [85]. This preliminary partial success of aramchol provided a rationale for SCD1 modulation, and aramchol is being further evaluated as a candidate drug for the treatment of NAFLD in an ongoing phase 3 trial.

Fibroblast growth factors 19 and 21 (FGF19 and FGF21) are newly discovered endocrine messengers that regulate multiple aspects of energy homeostasis. In particular, FGF21 has been shown to improve weight loss, lipid metabolism, and insulin resistance, and a phase 3 study of the application of an FGF21 analogue, pegbelfermin [86], in NAFLD treatment is ongoing. Similarly, a humanized monoclonal antibody agonist of the β-Klotho/FGFR1c receptor complex, MK-3655 (ClinicalTrials.gov identifier: NCT04583423), has been tested in a phase 2 study. MK-3655 binds to a unique epitope of β-Klotho, resulting in selective activation of FGFR1c signaling via the metabolic pathway utilized by FGF21-based ligand therapies.

**Table 1:** Potential agents for the treatment of NAFLD.

	Pharmacological Agent	Target	Trial Phase
1	Obeticholic acid	FXR agonist	3
2	Semaglutide	GLP-1 receptor agonist	3
3	Resmetirom (MGL-3196)	THR-β agonist	3
4	Aramchol	SCD1 inhibitor	3
5	Lanifbranor	PPARα/δ/γ agonist	3
6	Belapectin (GR-MD-02)	Galectin-3 inhibitor	3
7	Pegbelfermin (BMS-986036)	FGF21 analogue	3
8	MK-3655	Monoclonal antibody agonist of the b-Klotho/FGFR1c receptor complex	2
9	TVB-2640	FASN inhibitor	2
10	Tirzepatide	Dual GIP and GLP-1 receptor agonist	2
11	BI456906	Dual GIP and GLP-1 receptor agonist	2
12	BMS-986263	HSP47 siRNA	2
13	CC-90001	JNK inhibitor	2

FXR, farnesoid X receptor; GLP-1, glucagon-like peptide-1; THR-β, thyroid hormone receptor-β; SCD1, stearoyl-CoA desaturase 1; PPAR, peroxisome proliferative activated receptor; FGF, fibroblast growth factor; FGFR, FGF receptor; FASN, fatty acid synthase; GIP, glucose-dependent insulinotropic polypeptide; HSP, heat shock protein; JNK, Jun N-terminal kinase. This table does not cover all of the trials. In addition to the trials presented in this table, there are ongoing trials of GLP-1 receptor agonists, SGLT2 inhibitors, PPAR agonists, and combinations of a variety of drugs. (Adapted with permission from Ref. [87] 2022, Kyoto Prefectural University of Medicine.)

The HSP47 siRNA BMS-986263 [88] and the JNK inhibitor CC-90001 (ClinicalTrials.gov identifier: NCT04048876; recruitment status indicated that it is terminated (business objectives have changed)) have been evaluated for their therapeutic effects on liver fibrosis in NAFLD.

Galectins are markedly increased in inflammation, fibrosis, and cancer and are involved in the pathogenesis of these diseases, and they represent candidate damage-associated molecular patterns. Galectin-3 is the most prominent galectin. It is secreted mainly by macrophages in disease states, and galectin-3-deficient mice are protected from NASH progression and liver fibrosis. A galectin-3 inhibitor, belapectin [89], exhibited a significant effect on portal hypertension and the development of varices in a subgroup of patients without varices at baseline. A phase 3 study to evaluate the safety and efficacy of this drug in patients with NASH cirrhosis without esophageal varices is ongoing.

An inhibitor of fatty acid synthase, TVB-2640 [90], was found to reduce excess liver fat and to inhibit inflammation and fibrogenesis in the phase 2 trial FASCINATE-1, and a phase 3 study is ongoing.

Antagonists of toll-like receptor 4 (ClinicalTrials.gov identifier: NCT02442687) and mineralocorticoid receptor (ClinicalTrials.gov identifier: NCT02923154) and inhibitors of semicarbazide-sensitive amine oxidase/vascular adhesion protein 1 (ClinicalTrials.gov identifier: NCT03166735) and PNPLA3 (ClinicalTrials.gov identifier: NCT04483947) have been proposed to have potential efficacy in NAFLD. While no clinical trials of such agents have yet been officially reported, such trials may be initiated in the future.

### 6.2. Drugs with Suspended or Discontinued Development

Other drugs initially expected to have potential for the treatment of NAFLD or fibrosis have failed to reach the primary endpoints in clinical trials. Examples of such drugs include the PPARα/δ agonist elafibranor (ClinicalTrials.gov identifier: NCT02704403), the CCR2/5 antagonist cenicriviroc [91], the ASK1 inhibitor selonsertib [92], and the caspase inhibitor emricasan [93]. After treatment with the PPARδ agonist seladelpar (MBX-8025) (ClinicalTrials.gov identifier: NCT03551522), liver biopsy samples exhibited interface hepatitis in a phase 2 NASH trial (Table 2).

Additional drugs whose development has been suspended or discontinued are noted in Table 2. In particular, apararenone (MT-3995 [94]), which is an agonist of the non-steroidal mineralocorticoid receptor, was histologically found to improve multiple potential markers of fibrosis and to have anti-inflammatory and antifibrotic effects. However, there seem to be no plans for a phase 3 trial at this time. Similarly, aldafermin [95], an engineered FGF19 analog, reduced liver fat content and tended to improve fibrosis in a phase 2 trial of patients with NASH, but the primary endpoint was not reached. Finally, while lysyl oxidase-like 2 plays a central role in fibrogenesis by catalyzing the cross-linkage of collagen and elastin, simtuzumab [96], an antibody against this enzyme, was ineffective at decreasing hepatic collagen content.

Notably, it is possible that the clinical trials evaluating the drugs mentioned above will be modified in their design or will be re-scheduled. There are also plans for trials evaluating combinations of the drugs noted in Table 2 or agents modulating FXR, diacylglycerol acyltransferase 1/2, ACC acetyl-CoA carboxylase, FGF21, liver X receptor, and mitochondrial pyruvate carrier with anti-diabetic drugs, such as GLP-1 receptor agonists and SGLT2 inhibitors. Although the current landscape of drug development is complex, it is clear that many clinical trials and plans for clinical trials are underway to establish additional effective treatments for NAFLD.

## 7. Conclusions

In this review, we explored the potential mechanisms, particularly the role of hepatic stellate cells, in the progression of NAFLD, and the current knowledge of candidate therapeutic agents, including natural products. A comprehensive understanding of these aspects will pave the way to preventing the onset and progression of NAFLD. Liver fibrosis is the factor most commonly associated with all-cause mortality among patients with NAFLD. No FDA-approved anti-NAFLD drugs are currently available; therefore, it is important to establish therapeutic agents and preventive interventions with the potential to combat fibrosis and reduce the risk of hepatocarcinogenesis. Long-term administration of safe and cost-effective drugs is used for the management of NASH, the necroinflammatory form of NAFLD, and liver fibrosis, which are considered the main targets. HNK has been widely used as a component of herbal drugs in daily practice. It reportedly possesses anti-tumor properties and has an inhibitory effect on fibrosis progression; thus, it has the therapeutic potential for clinical prevention and treatment of liver fibrosis and liver cancer. Importantly, a variety of candidate drugs for the treatment of NAFLD are currently being tested in phase II or III clinical trials, and some have demonstrated efficacy toward steatosis, inflammation, and fibrosis. The results of these clinical trials are awaited.

## Figures and Tables

**Table 2:** Drugs with suspended or discontinued development.

	Pharmacological Agent	Target	Trial Phase
1	Elafibranor	PPARα/δ agonist	3
2	Cenicriviroc	CCR2/5 antagonist	3
3	Selonsertib	ASK1 inhibitor	3
4	Seladelpar (MBX-8025)	PPARδ agonist	2
5	Emricasan	Caspase inhibitor	2
6	Apararenone (MT-3995)	Non-steroidal MR antagonist	2
7	Aldafermin (NGM-282)	FGF19 analogue	2
8	Simtuzumab	Anti-LOXL2 antibody	2
	**Reasons for discontinuation**		
	1 Elafibranor failed to reach the primary endpoint in a phase 3 trial.		
	2 A phase 3 trial of cenicriviroc was terminated early due to lack of efficacy.		
	3 Selonsertib failed to reach the primary endpoint in a phase 3 trial.		
	4 Seladelpar showed signs of significant adverse effects in a phase 2 trial.		
	5 Emricasan failed to reach the primary endpoints in a phase 2 trial.		
	6 There are no plans for a phase 3 trial of apararenone at this time.		
	7 Aldafermin (NGM282) failed to reach the primary endpoint in a phase 2 trial.		
	8 Simtuzumab failed to reach the primary endpoints in a phase 2 trial.		

PPAR, peroxisome proliferative activated receptor; CCR2/5, C-C chemokine receptor type 2/5; ASK1, apoptosis signal-regulating kinase 1; MR, mineralocorticoid receptor; FGF, fibroblast growth factor; LOXL2, lysyl oxidase-like 2. (Adapted with permission from Ref. [87] 2022, Kyoto Prefectural University of Medicine.)
